# Genomic prediction of carcass traits using different haplotype block partitioning methods in beef cattle

**DOI:** 10.1111/eva.13491

**Published:** 2022-11-14

**Authors:** Hongwei Li, Zezhao Wang, Lei Xu, Qian Li, Han Gao, Haoran Ma, Wentao Cai, Yan Chen, Xue Gao, Lupei Zhang, Huijiang Gao, Bo Zhu, Lingyang Xu, Junya Li

**Affiliations:** ^1^ Laboratory of Molecular Biology and Bovine Breeding, Institute of Animal Sciences Chinese Academy of Agricultural Sciences Beijing China

**Keywords:** Bayesian models, beef cattle, GBLUP, haplotype, linkage disequilibrium

## Abstract

Genomic prediction (GP) based on haplotype alleles can capture quantitative trait loci (QTL) effects and increase predictive ability because the haplotypes are expected to be in linkage disequilibrium (LD) with QTL. In this study, we constructed haploblocks using LD‐based and the fixed number of single nucleotide polymorphisms (fixed‐SNP) methods with Illumina BovineHD chip in beef cattle. To evaluate the performance of different haplotype block partitioning methods, we constructed haploblocks based on LD thresholds (from *r* ^2^ > 0.2 to *r* ^2^ > 0.8) and the number of fixed‐SNPs (5, 10, 20). The performance of predictive methods for three carcass traits including liveweight (LW), dressing percentage (DP), and longissimus dorsi muscle weight (LDMW) was evaluated using three approaches (GBLUP and BayesB model based on the SNP, G_H_BLUP, and BayesBH models based on the haploblock, and G_H_BLUP+GBLUP and BayesBH+BayesB models based on the combined haploblock and the nonblocked SNPs, which were located between blocks). In this study, we found the accuracies of LD‐based and fixed‐SNP haplotype Bayesian methods outperformed the Bayesian models (up to 8.54 ± 7.44% and 5.74 ± 2.95%, respectively). G_H_BLUP showed a high improvement (up to 11.29 ± 9.87%) compared with GBLUP. The Bayesian models have higher accuracies than BLUP models in most scenarios. The average computing time of the BayesBH+BayesB model can reduce by 29.3% compared with the BayesB model. The prediction accuracies using the LD‐based haplotype method showed higher improvements than the fixed‐SNP haplotype method. In addition, to avoid the influence of rare haplotypes generated from haplotype construction, we compared the performance of GP by filtering four types of minor haplotype allele frequency (MHAF) (0.01, 0.025, 0.05, and 0.1) under different conditions (LD levels were set at *r* ^2^ > 0.3, and the fixed number of SNPs was 5). We found the optimal MHAF threshold for LW was 0.01, and the optimal MHAF threshold for DP and LDMW was 0.025.

## 1 INTRODUCTION

Genetic improvement for carcass traits is greatly important in the beef cattle breeding industry. Single nucleotide polymorphisms (SNP) are routinely applied to increase the prediction accuracy at a young age compared with the approach using pedigree information in farm animals (VanRaden, 2008). A haplotype block (haploblock) defines a region of the genome, comprising a set of neighboring SNPs whose alleles are likely to be inherited together (Hess et al., 2017). Haplotypes have been previously proposed in human genetics (Chapman et al., 2003; Curtis, 2007; Curtis et al., 2001; Gabriel et al., 2002; North et al., 2006), and this strategy was widely used for the prediction of genomic breeding values in animal breeding studies (Calus et al., 2008, 2009; Cuyabano et al., 2014; Cuyabano, Su, & Lund, 2015; Hess et al., 2017; Li et al., 2021; Villumsen & Janss, 2009). However, the prediction accuracy can be influenced by the number of neighboring SNPs contained in a haploblock and the length of the haploblock. When the boundaries of haploblocks are determined appropriately, the haplotype‐based methods can produce high accuracy over SNP‐based methods for both real (Cuyabano, Su, Rosa, et al., 2015; Hess et al., 2017) and simulated data (Villumsen et al., 2009).

In general, the methods for constructing haploblocks can be divided into two types, including the fixed‐length haplotypes and the variable‐length haplotypes. The fixed‐length haploblock methods include the fixed‐SNP number (Calus et al., 2009; Hayes et al., 2007; Villumsen et al., 2009) and the fixed length (Hess et al., 2017) in a block, this method is easy to implement and costs less time; however, the population‐specific linkage disequilibrium (LD) information were not included, which may greatly influence the haploblock construction. A variable‐length method was proposed to obtain co‐segregation of allele combinations based on LD information within the population, which can result in a decrease in the number of haplotype alleles (Rinaldo et al., 2005).

The accuracy of GP can be affected by the population size, the relationships of individuals, and the phasing approach. Determination of the haplotype phase is important in the development of large‐scale sequencing (Browning & Browning, 2011). Many programs, such as PHASE (Crawford et al., 2004), BEAGLE (Browning & Browning, 2009), DAGPHASE (Druet & Georges, 2010), SHAPEIT (O'Connell et al., 2014), and LINKPHASE3 (Druet & Georges, 2015) were proposed and compared in previous studies (Choi et al., 2018). Among them, the BEAGLE (Browning & Browning, 2009) were expected to obtain a high accuracy as they constructed haplotype based on population‐based constructs by including the related individuals with more shared haplotype (Ferdosi et al., 2016).

A recent study reported the LD‐based haplotype methods can increase by 3.1% in the Nordic Holstein population compared with the individual SNP approach (Cuyabano et al., 2014). Hess et al. (2017) found GP based on fixed‐length haplotype alleles rather than SNPs can increase the accuracy of genomic prediction (GP) up to 5.5%. The haplotype methods performed better than SNP methods in GP and are less sensitive to prior and hyperparameter settings (Villumsen & Janss, 2009). Previous studies mainly focused on the estimation of prediction accuracy using a single haploblock construction method. However, the accuracy of GP for economically important traits using different haploblock methods has not been fully explored and compared in beef cattle.

The objectives of the current study were to (1) assess the performance of GP for carcass traits and computation time based on different haploblock methods (the fixed‐SNPs and the LD‐based) using GBLUP and BayesB model; (2) compare the prediction accuracies under different LD thresholds and different number of SNPs; (3) evaluate the prediction accuracy with different MHAF thresholds for three traits.

## 2 MATERIALS AND METHODS

### 2.1 Data

The phenotypic data were generated from 1478 Chinese Simmental cattle, which were born between 2008 and 2020 from Ulgai, Xilingol League, and Inner Mongolia, China. After weaning, all individuals were moved to Jinweifuren Co., Ltd. for fattening under the same feeding and management conditions. All individuals were slaughtered at an average age of 20 ± 2.2 months. More detailed description of the management processes was reported in previous studies (Zhu et al., 2016, 2017). Carcass traits were measured by the guidelines proposed by the Institute of Meat Purchase Specifications established by the Agricultural Marketing Service (AMS) of the USDA. Liveweight (LW), dressing percentage (DP), and longissimus dorsi muscle weight (LDMW) were selected for subsequent analysis. After removing missing values and outliers, the basic statistics of phenotype data were shown in Table 1.

**TABLE 1:** Statistical description and heritability estimation for three carcass traits

Trait	Number of phenotypes	Mean ± SD	Maximum	Minimum	*h* ^2^ ± SE
LW	1387	518.55 ± 80.95	790.00	318.00	0.40 ± 0.06
DP	1366	0.54 ± 0.03	0.62	0.45	0.22 ± 0.06
LDMW	1457	32.78 ± 8.35	59.00	9.89	0.16 ± 0.05

Abbreviations: DP, dressing percentage; LDMW, longissimus dorsi muscle weight; LW, liveweight.

### 2.2 Genotyping and phasing

The DNA was extracted from blood samples. The Illumina BovineHD BeadChips (777,962 SNPs) were used for genotyping. Before phasing the quality control of the original SNP dataset was carried out using PLINK (v1.07) (Chang et al., 2015; Purcell et al., 2007). Individuals and autosomal SNPs were filtered by the following criteria, SNPs call rate (<0.90), minor allele frequency (MAF <0.01), Hardy–Weinberg Equilibrium (*p* < 10^−5^), and individual call rate (<0.90). Missing genotypes were imputed using BEAGLE (v4.1) (Browning & Browning, 2016). Consequently, 1478 individuals and 672,060 SNPs remained and further phased using BEAGLE (v4.1) (Browning & Browning, 2016) with default parameters.

### 2.3 Heritability and variance component estimation

Multivariate variance analysis was used to determine the fixed effects of sex, year, fattening farm, and slaughterhouse and the covariates (body weight upon entering the fattening farm and the number of fattening days). The phenotypes were adjusted by the significant factors using glm function in R programming. Variance components were estimated using the following univariate animal model in ASREML (v4.1).

(1) $$y=1_{n}\mu +\mathrm{Za}+e,$$

where y is the vector of the adjusted phenotypes, $1_{n}$ is an n×1 vector with entries equal to 1; *μ* is the overall mean; $a∼N\left(0,\sigma_{a}^{2}G\right)$ is a vector of random additive genetic effect, where G is the additive genomic relationship matrix constructed using all SNPs, and $\sigma_{a}^{2}$ is the additive genetic variance, Z is the incidence matrix linking a to y; and $e∼N\left(0,\sigma_{e}^{2}I\right)$ is a vector of random residuals, where I is the identity matrix, and $\sigma_{e}^{2}$ is the residual variance. The heritability was estimated as *h* ^2^ $=\sigma_{a}^{2}$/(${\sigma_{a}^{2}+\sigma }_{e}^{2}$).

### 2.4 Haplotype construction

#### 2.4.1 LD‐based method

The LD information was used to determine the boundary of haploblocks for each chromosome. Due to the variation in local recombination rates, the breakdown of LD is often discontinuous and presents a haploblock‐like structure (Daly et al., 2001). Therefore, it is important to analyze the haploblock structure and haplotypes that underlie LD.

A group of SNPs was defined as a haploblock if the LD between every two consecutive SNPs in the group was greater than or equal to the square of correlation coefficient (*r* ^2^). There were no overlapping SNPs between adjacent haploblock. For two bi‐allelic loci ($A_{1}/A_{2}$ and $B_{1}/B_{2}$), *r* ^2^ was calculated as

(2) $$r^{2}=\frac{D^{2}}{\left(p_{A_{1}}p_{A_{2}}p_{B_{1}}p_{B_{2}}\right)},$$

where $D=p_{A_{1}B_{1}}p_{A_{2}B_{2}}-p_{A_{1}B_{2}}p_{A_{2}B_{1}}$. D is the degree of LD for bi‐allelic loci ($A_{1}/A_{2}$ and $B_{1}/B_{2}$); $p_{A_{1}}$, $p_{A_{2}}$, $p_{B_{1}}$, and $p_{B_{2}}$ are the alleles ($A_{1}$, $A_{2}$, $B_{1}$, and $B_{2}$) frequencies; $p_{A_{1}B_{1}}$, $p_{A_{2}B_{2}}$, $p_{A_{1}B_{2}}$, and $p_{A_{2}B_{1}}$ is the genotype ($A_{1}B_{1}$, $A_{2}B_{2}$, $A_{1}B_{2}$, and $A_{2}B_{1}$) frequencies (Hill, 1981; Hill & Robertson, 1968).

Seven different LD levels (*r* ^2^ > 0.2, 0.3, 0.4, 0.5, 0.6, 0.7, and 0.8) were set as the thresholds in this study. To reduce the dimensionality of LD‐based haplotype analyses, haplotype alleles with a minor allele frequency of less than 0.01 were discarded.

#### 2.4.2 Fixed‐SNP method

The fixed‐SNP haplotype was defined by the fixed number of SNPs in one haploblock (e.g., 5‐SNPs per haploblock). Three different levels (5‐, 10‐, and 20‐SNPs) were set to construct the haploblock. There were no overlapping SNPs between adjacent haploblocks. Consistent with the LD method, haplotype alleles with a minor allele frequency of less than 0.01 were discarded in the current study.

#### 2.4.3 MHAF thresholds set

To compare the haplotype structure distribution and performance of GP with different MHAF thresholds, the LD levels were set at *r* ^2^ > 0.3 and the fixed number of SNPs was 5, while the quality control of haplotype alleles was set at four different levels (0.01, 0.025, 0.05 and 0.1).

### 2.5 GP models

Haplotype effects were modeled using numerical dosage coding strategies (Calus et al., 2008; Cuyabano et al., 2014; Cuyabano, Su, & Lund, 2015; Da, 2015; Meuwissen et al., 2014). A group of SNPs was contained in a haploblock. In the numerical dosage model, the SNPs were regarded as a unit and coded as the number of copies, which were termed as the haplotype alleles. The GP was performed using the GBLUP model and BayesB model, fitting covariates for either SNPs or haplotype alleles. The approaches include GBLUP and BayesB model (only based on the SNPs), G_H_BLUP and BayesBH model (only based on haploblock), and G_H_BLUP+GBLUP and BayesBH+BayesB model (contain both the haploblock and the nonblocked SNPs) were considered for predictions. Seven different *r* ^2^ thresholds and three levels of a fixed number of SNPs were used for haploblock construction. The Bayesian algorithm was performed using GCTB (v2.0) (Zeng et al., 2018), a single Markov‐chain Monte Carlo (MCMC) with a length of 30,000, while the first 10,000 cycles were discarded as the burn‐in period.

#### 2.5.1 GBLUP model

The GBLUP model (including only the SNP effect) was used for LW, DP, and LDMW as described in Equation (1).

We performed GP using GBLUP for all SNPs, and the genomic relationship matrix was calculated as $G=\frac{\left(M-P\right){\left(M-P\right)}^{\prime }}{m\left[2p_{i}\left(1-p_{i}\right)\right]}$, where *M* means the (0, 1, 2)‐encoded genotype matrix, $p_{i}$ is the MAF of marker i, m is the number of markers, and P is a matrix with columns equal to $2p_{i}$ (Amin et al., 2007; Leutenegger et al., 2003).

The haplotype‐based genomic best linear unbiased prediction (G_H_BLUP) was performed for all SNPs. The haplotype‐based genomic relationship matrix $G_{H}$ was expressed as $G_{H}=\frac{\left(M_{H}-P_{H}\right){\left(M_{H}-P_{H}\right)}^{\prime }}{m_{H}\left[2p_{i}\left(1-p_{i}\right)\right]}$, where $M_{H}$ is the pseudo‐markers matrix with entries 0, 1, and 2 representing the number of copies of each haplotype allele in a haploblock, and $p_{i}$ is the MAF of haplotype alleles i, $m_{H}$ is the number of haplotype alleles of the whole genome, and $P_{H}$ is a matrix with columns equal to $2p_{i}$. In the G_H_BLUP+GBLUP model:

(3) $$y=1_{n}\mu +\mathrm{Za}+Z_{u}a_{u}+e,$$

which included the haploblock effects and the SNP effects estimated from outside the haploblocks (nonblocked SNPs). $a∼N\left(0,\sigma_{a}^{2}G_{H}\right)$ is a vector of random additive genetic effect, where $G_{H}$ is the additive genetic relationship matrix constructed using haplotype alleles and $\sigma_{a}^{2}$ is the additive genetic variance based on the haplotype alleles, Z is the incidence matrix associating a; $a_{u}∼N\left(0,\sigma_{a_{u}}^{2}G\right)$ is a vector of random additive genetic effect, where G is the additive genetic relationship matrix constructed using nonblocked SNPs and $\sigma_{a_{u}}^{2}$ is the additive genetic variance based on the nonblocked SNPs, $Z_{u}$ is incidence matrix associating $a_{u}$; a is composed of haplotype alleles effects and $a_{u}$ is composed of SNP effects estimated from outside the haploblocks.

#### 2.5.2 BayesB model

The BayesB, BayesBH, and BayesBH+BayesB models were defined by the Equation (4)

(4) $$y=u+\sum_{j=1}^{M}Z_{j}\alpha_{j}\delta_{j}+e,$$

where y is the vector of the adjusted phenotypes; u denotes the overall mean; M is the number of covariates for SNPs (BayesB model) or haplotype alleles (BayesBH model) or SNPs and haplotype alleles (BayesBH+BayesB model), $Z_{j}$ is a vector of alleles counts (0/1/2) at SNP j or haplotype allele j, BayesB assumes that some of the $\alpha_{j}$ have no effect. This is defined by the binary variable $\delta_{j}$ that represents whether covariate j was fitted in the model according to hyperparameter π, such that δ=1 with probability 1−π, or δ=0 with probability π. In this study, π was set to 0.96, 0.995, and 0.995 for LW, DP, and LDMW, respectively, according to the previous studies (Wang et al., 2019). $e∼N\left(0,\sigma_{e}^{2}\right)$ is a vector of residual effects.

### 2.6 Evaluation of prediction performance

Both the accuracy and bias of GP were assessed based on 5‐fold cross‐validation (CV). The CV procedure is that the whole population was randomly divided into 5 groups, one of which was taken as the validation group each time, and the remaining 4 groups were used as the reference group. This procedure was randomly repeated 5 times for GBLUP and Bayesian models, respectively. The prediction accuracy is the average Pearson correlation coefficient between the adjusted phenotypic values and genomic estimated breeding values (GEBVs) in the validation groups (Bolormaa et al., 2013). The formula is as follows,

(5) $$\text{Prediction accuracy}=\mathrm{cor}\left(y,\text{gebv}\right),$$

where y is the vector of the adjusted phenotypes, and gebv is the vector of the GEBVs.

The regression coefficient of the adjusted phenotype on GEBVs for individuals in the validation group was obtained to measure the degree of inflation/deflation of prediction and was defined as follows,

(6) $$b=\frac{\mathrm{cov}\left(\text{gebv},y\right)}{\mathrm{var}\left(\text{gebv}\right)},$$

where y and gebv is the same as that in Equation (5), the regression coefficient of unbiased models is expected to be close to 1, whereas values greater than 1 indicate a biased deflation prediction of GEBVs, and values smaller than 1 indicate a biased inflation prediction of GEBVs (Xu et al., 2020).

To determine whether haplotype methods can significantly improve prediction accuracy over the SNP methods, we used the one‐sided paired *t‐*test to examine the significance of the differences. The level of statistical significance was set at *p* < 0.05.

## 3 RESULTS

### 3.1 Heritability estimation and haploblock construction

As shown in Table 1, the estimated heritability of LW, DP, and LDMW using the univariate animal model were 0.40, 0.22, and 0.16, respectively. The statistical description of haploblock structure was shown in Table 2. Notably, we observed the number of haploblocks decreased with LD level (from 68,345 to 42,097), while the number of haplotype alleles decreased from 1,010,588 to 747,477. Under different LD levels (from *r* ^2^ > 0.2 to *r* ^2^ > 0.8), the average number of SNPs within each haploblock ranged from 3.53 to 5.17, while the maximum number of SNPs within each haploblock was from 53 to 99.

**TABLE 2:** Haploblock structure of different haploblock construction methods

Method	*r* ^2^ ^a^/Number of SNPs^b^	Number of haploblocks	Number of SNPs in blocks	Number of SNPs out of blocks	Number of SNPs per haploblock		Number of alleles
					Mean	Max	
LD	0.2	68,345	353,356	318,704	5.17	99	1,010,588
	0.3	64,765	300,455	371,605	4.64	99	914,760
	0.4	60,471	260,530	411,530	4.31	88	856,417
	0.5	56,035	227,982	444,078	4.07	88	817,085
	0.6	51,704	199,825	472,235	3.86	85	788,801
	0.7	47,134	174,074	497,986	3.69	53	766,424
	0.8	42,097	148,271	523,789	3.52	53	747,477
Fixed‐SNPs	5	134,412	672,060	0	5	5	1,526,223
	10	67,206	672,060	0	10	10	2,122,577
	20	33,603	672,060	0	20	20	2,950,340

Abbreviations: Fixed‐SNPs, fixed‐SNPs Haploblock construction method; LD, LD‐based Haploblock construction method; LD, linkage disequilibrium; SNP, single nucleotide polymorphisms.

^a^ The seven different LD thresholds set from *r* ^2^ > 0.2 to *r* ^2^ > 0.8 to construct LD‐based haploblocks.

^b^ The three levels of the number of SNPs (5, 10, and 20) to construct fixed‐SNP haploblocks.

As for the fixed‐SNP method, the number of haploblocks was from 33,603 to 134,412, while the number of fixed‐SNPs was between 5 and 20. Details about haploblocks (the total number of haploblocks, the number of SNPs in blocks, the number of SNPs out of blocks, the number of SNPs within haploblock with different *r* ^2^, and the number of fixed‐SNPs) were presented in Table 2.

Moreover, under the strict MHAF thresholds (from 0.01 to 0.1), the number of haplotype alleles based on the haplotype‐based method with *r* ^2^ > *r* ^2^ > 0.3 (0.3LD) decreased from 605,908 to 403,841, while the number of fixed‐SNPs were 5 (5‐SNPs) decreased from 755,697 to 382,073. The number of alleles based on the two methods decrease by 33.35% and 49.44%. Our results revealed that 5‐SNP methods contain more alleles with low MHAF. Details about haplotype structure at different MHAF thresholds were shown in Table 3.

**TABLE 3:** The haplotype structure at different MHAF thresholds

MHAF	Number of alleles	Number of haplotype alleles	Number of SNPs out of blocks	Number of haploblocks
SNP	672,060	—	—	—
0.3LD	914,760	543,155	371,605	64,765
0.3LD_MAF_0.01^a^	605,908	235,608	370,300	63,996
0.3LD_MAF_0.025	526,708	189,640	337,068	63,330
0.3LD_MAF_0.05	472,152	163,405	308,747	61,417
0.3LD_MAF_0.1	403,841	134,864	268,977	58,415
5‐SNPs	1,526,223	1,526,223	—	134,412
5‐SNPs_MAF_0.01^b^	755,697	755,697	—	134,388
5‐SNPs_MAF_0.025	601,497	601,497	—	134,297
5‐SNPs_MAF_0.05	498,006	498,006	—	133,976
5‐SNPs_MAF_0.1	382,073	382,073	—	132,869

Abbreviations: MHAF, minor haplotype allele frequency; SNP, single nucleotide polymorphisms.

^a^ 0.3LD_MAF_0.01: Haplotype alleles with a minor allele frequency less than 0.01 based on the 0.3LD haplotype construction method were discarded.

^b^ 5‐SNPs_MAF_0.01: Haplotype alleles with a minor allele frequency less than 0.01 based on the 5‐SNP haplotype construction method were discarded.

### 3.2 Performance of GP

#### 3.2.1 Performance of GP based on different models

**FIGURE 1:**
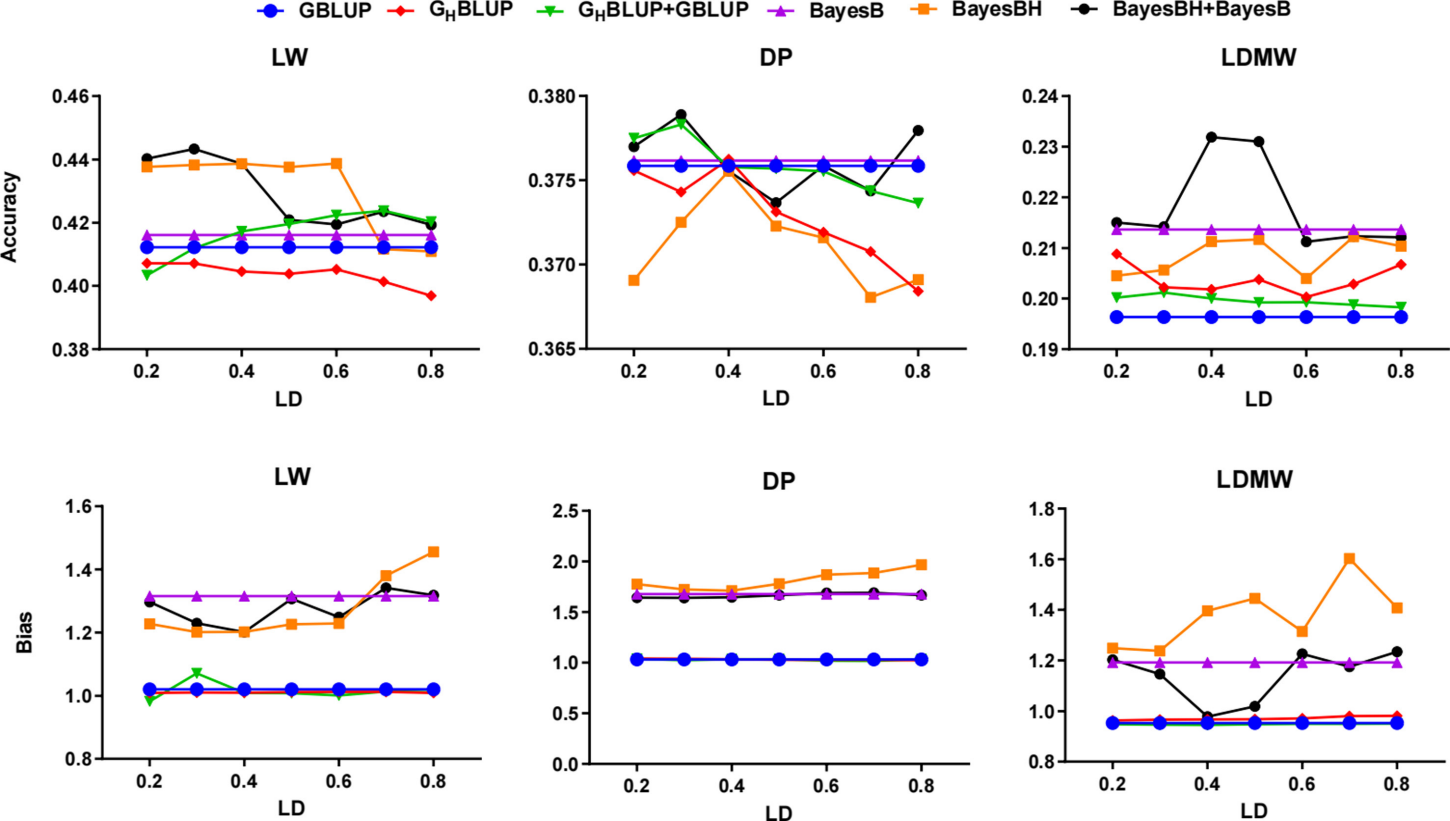
Prediction accuracies and bias of different *r* ^2^ thresholds for three traits based on the SNP and haplotype alleles using GBLUP and BayesB models. DP, dressing percentage; LD, linkage disequilibrium; LDMW, longissimus dorsi muscle weight; LW, liveweight; SNP, single nucleotide polymorphisms.

To assess the performances of GP, we compared six models (GBLUP, G_H_BLUP, G_H_BLUP+GBLUP, BayesB, BayesBH, and BayesBH+BayesB) using different haplotype construction methods. In this study, our results revealed the accuracies of GP decreased with the trait heritability. The accuracies of haplotype methods showed great improvement for all three traits under some LD compared with the GBLUP model. Among these traits, we observed the highest accuracies for LW, followed by DP and LDMW (Figure 1). Compared with the GBLUP model, the G_H_BLUP showed ~6.35 ± 9.39% and ~ 11.29 ± 9.87% higher average accuracy for LDMW using LD‐based and fixed‐SNP method (Tables 4 and 5), while the G_H_BLUP+GBLUP showed ~1.1 ± 7.3% higher average accuracy for LW across all LD levels (Table 4). The Bayesian models have high accuracies in most scenarios compared with BLUP models. Among the six models, BayesBH+BayesB had the best performance for all three traits (Figure 1) and showed increasing accuracy of GP for LDMW (up to 8.54% higher than SNP models) (Table 4). However, as for DP and LDMW, BayesBH model cannot improve the accuracy and have larger biases compared with the SNP‐based method in most scenarios.

**TABLE 4:** Percent change of predictive accuracies using the LD‐based haplotype model rather than SNPs for three traits in Chinese Simmental beef cattle (±SE)

	Trait	LD‐based method^a^							
		0.2	0.3	0.4	0.5	0.6	0.7	0.8	Mean
G_H_BLUP	LW	−1.24 ± 6.26	−1.26 ± 6.20	−1.89 ± 6.19	−2.09 ± 6.19	−1.74 ± 6.06	−2.681 ± 5.96	−3.83 ± 5.83	−2.1 ± 6.1
	DP	−0.073 ± 6.71	−0.41 ± 6.77	0.10 ± 6.91	−0.73 ± 6.89	−1.06 ± 6.9	−1.367 ± 6.88	−2.008 ± 6.81	−0.79 ± 6.84
	LDMW	6.35 ± 9.39*	2.83 ± 9.06*	2.71 ± 9.03*	3.68 ± 9.14	1.96 ± 8.89	3.251 ± 8.83	5.12 ± 8.71*	3.7 ± 9.01
G_H_+G	LW	−2.25 ± 7.86	−0.086 ± 9.10	1.21 ± 7.11	1.76 ± 6.97	2.41 ± 6.86	2.72 ± 6.64*	1.90 ± 6.54	1.1 ± 7.3
	DP	0.44 ± 6.51	0.65 ± 6.49	−0.019 ± 6.55	−0.043 ± 6.56	−0.088 ± 6.53	−0.395 ± 6.57	−0.59 ± 6.70	−0.01 ± 6.56
	LDMW	1.85 ± 9.34*	2.42 ± 9.33**	1.83 ± 9.21**	1.44 ± 9.13**	1.48 ± 9.11**	1.224 ± 9.07**	0.97 ± 9.02**	1.6 ± 9.17
BayesBH	LW	5.18 ± 2.94**	5.31 ± 2.97**	5.42 ± 3.04**	5.16 ± 2.95**	5.43 ± 3**	−1.08 ± 3.02	−1.26 ± 3.06	3.45 ± 3
	DP	−1.89 ± 5.47	−0.97 ± 5.56	−0.17 ± 5.46	−1.03 ± 5.51	−1.21 ± 5.54	−2.16 ± 5.54	−1.88 ± 5.5	−1.33 ± 5.51
	LDMW	−4.27 ± 6.04	−3.75 ± 6.7	−1.13 ± 6.4	−0.93 ± 6.48	−4.52 ± 6.23	−0.67 ± 6.4	−1.54 ± 6.39	−2.4 ± 6.38
BH+B	LW	5.81 ± 3.12**	6.53 ± 3.02**	5.41 ± 2.94**	1.15 ± 3.02	0.8 ± 2.93	1.79 ± 2.24	0.77 ± 2.96**	3.18 ± 2.89
	DP	0.22 ± 5.6	0.72 ± 5.64*	−0.16 ± 5.59	−0.66 ± 5.56	−0.08 ± 5.64	−0.48 ± 5.56	0.48 ± 5.64	0.01 ± 5.6
	LDMW	0.64 ± 6.59	0.25 ± 7.03	8.54 ± 7.44*	8.13 ± 7.29**	−1.13 ± 6.59	−0.64 ± 6.65	−0.73 ± 6.77	2.15 ± 6.91

Abbreviations: BayesBH, BayesB model based on haplotype; BH+B, BayesBH+BayesB model; DP, dressing percentage; G_H_+G, G_H_BLUP+GBLUP model; G_H_BLUP, GBLUP model based on haplotype; LD, linkage disequilibrium; LDMW, longissimus dorsi muscle weight; LW, liveweight; SNP, single nucleotide polymorphisms.

^a^ The seven different LD thresholds set from *r* ^2^ > 0.2 to *r* ^2^ > 0.8 to construct LD‐based haploblocks.

*Higher accuracy than the SNP model (*p* < 0.05); **Higher accuracy than the SNP model (*p* < 0.01).

**TABLE 5:** Percent change of predictive accuracies using fixed‐SNPs haplotype model rather than SNPs for three traits in Chinese Simmental beef cattle (±SE).

Model	Trait	Fixed‐SNP method^a^			
		5	10	20	Mean
G_H_BLUP	LW	1.09 ± 6.41*	1.03 ± 6.47	0.57 ± 6.5	0.9 ± 6.46
	DP	0.78 ± 6.68**	0.29 ± 6.59	0.07 ± 6.62	0.38 ± 6.63
	LDMW	10.52 ± 9.68**	11.29 ± 9.87**	10.24 ± 9.54**	10.68 ± 9.7
BayesBH	LW	5.74 ± 2.95**	1.03 ± 3	0.61 ± 3.05	2.46 ± 3
	DP	0.2 ± 5.49	−0.45 ± 5.44	−0.76 ± 5.37	−0.34 ± 5.43
	LDMW	3.61 ± 6.41	3.7 ± 6.62	3.65 ± 6.64	3.65 ± 6.56

Abbreviations: DP, dressing percentage; LDMW, longissimus dorsi muscle weight; LW, liveweight; SNP, single nucleotide polymorphisms.

^a^ The three levels of number of SNPs (5, 10, and 20) to construct fixed‐SNPs haplotype.

*Higher accuracy than the SNP model (*p* < 0.05); **Higher accuracy than the SNP model (*p* < 0.01).

#### 3.2.2 Accuracies and biases based on different LD levels

We constructed haploblocks and compared accuracies and bias based on different LD levels (from *r* ^2^ > 0.2 to *r* ^2^ > 0.8). Comparing with BayesB model, BayesBH+BayesB model with *r* ^2^ > 0.3 for LW (*h* ^2^ = 0.40) and DP (*h* ^2^ = 0.22) increased ~6.53 ± 3.02% and 0.72 ± 5.64%, while this model increase ~8.54 ± 7.44% for LDMW (*h* ^2^ = 0.16) under *r* ^2^ > 0.4 (Table 4). The BayesBH had a better performance for LW in most LD levels than the BayesB model (Figure 1; Table 4). G_H_BLUP models with *r* ^2^ > 0.2 for LDMW showed up to 6.35 ± 9.39% accuracy compared with the GBLUP model, while G_H_BLUP models for LW and DP did not increase the accuracies at all LD levels (Table 4). As shown in Figure 1, the biases of Bayesian models were larger than the GBLUP models for all three traits, while the biases of G_H_BLUP+GBLUP were similar to G_H_BLUP models (Figure 1; Table S3). In addition, the low accuracy and large bias of GP were found for LW and LDMW with the strict LD (Figure 1).

#### 3.2.3 Accuracies and biases of GP based on different numbers of fixed‐SNPs

**FIGURE 2:**
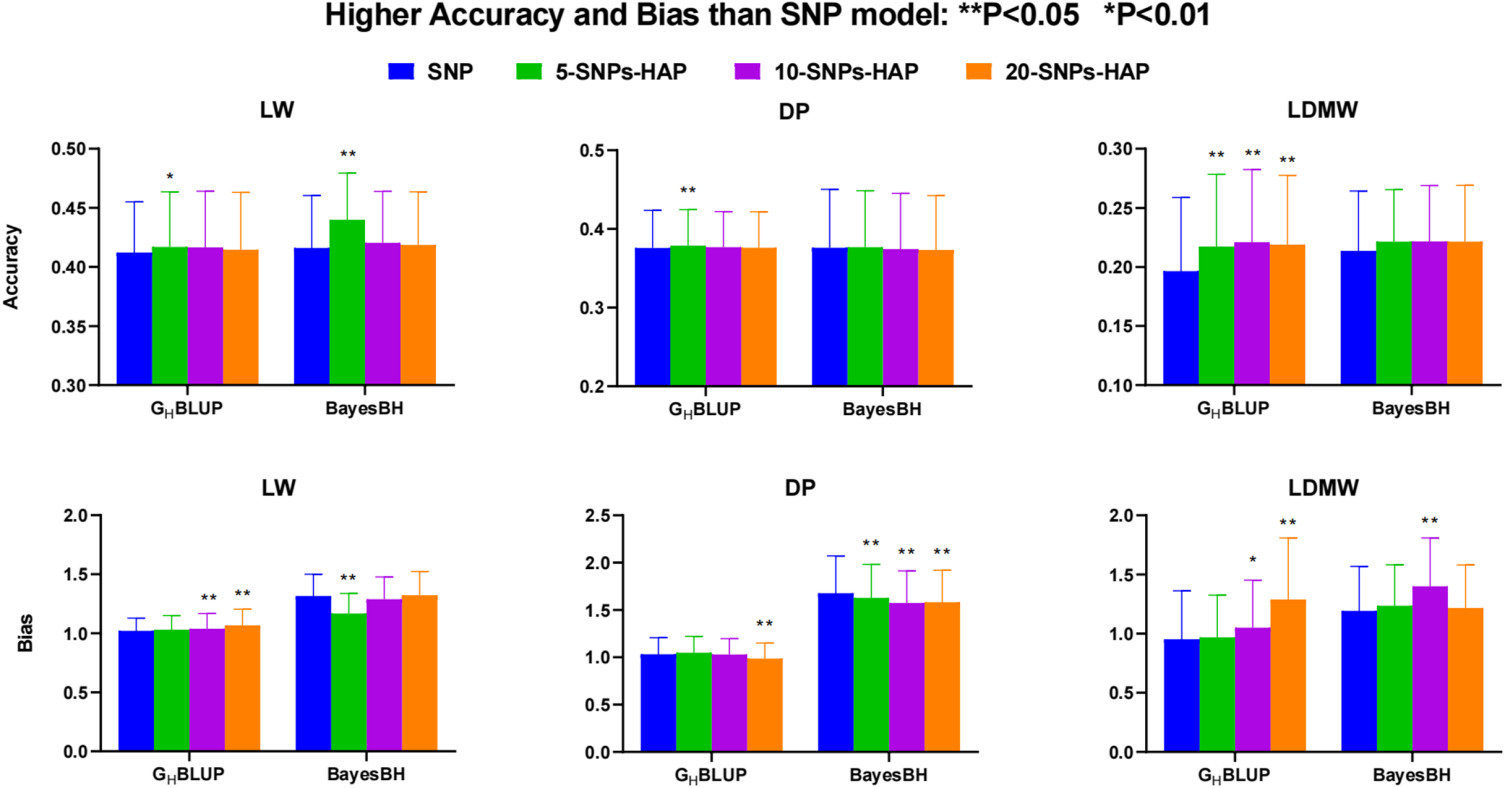
Prediction accuracies and bias of different number of SNPs in a block for three traits based on the SNP and haplotype alleles using GBLUP and BayesB models. DP, dressing percentage; LD, linkage disequilibrium; LDMW, longissimus dorsi muscle weight; LW, liveweight; SNP, single nucleotide polymorphisms.

To evaluate the accuracies of GP using the fixed‐SNP method, we set three different levels (5, 10, and 20‐SNPs) based on the number of fixed‐SNPs. Four models (GBLUP, G_H_BLUP, BayesB, and BayesBH) were used to investigate the performance of GP. We found that the 5‐SNPs G_H_BLUP model can significantly increase (*p* < 0.05) the accuracies of GP for all three traits compared with the GBLUP model, while the bias between G_H_BLUP based on fixed‐SNPs and GBLUP model significantly increased with the number of fixed‐SNPs (Figure 2; Table 5; Tables S2 and S3). As shown in Figure 2 and Table 5, all three fixed‐SNP BayesBH models (5‐SNPs BayesBH, 10‐SNPs BayesBH, 20‐SNPs BayesBH) tended to have higher accuracies than BayesB models, but the difference was not significant (*p* > 0.1), except for LW with 5‐SNPs BayesBH model (*p* < 0.001). Comparing with the BayesB model, the difference in accuracies of the 5‐SNPs BayesBH model was 5.74 ± 2.95%, 0.2 ± 5.49%, and 3.61 ± 6.41% for LW, LDMW, and DP, respectively (Table 5). Also, the 10‐SNPs G_H_BLUP model yielded ~11.29 ± 9.87% higher accuracy than the GBLUP model for LDMW.

#### 3.2.4 Comparison of performance of GP with different MHAF

**FIGURE 3:**
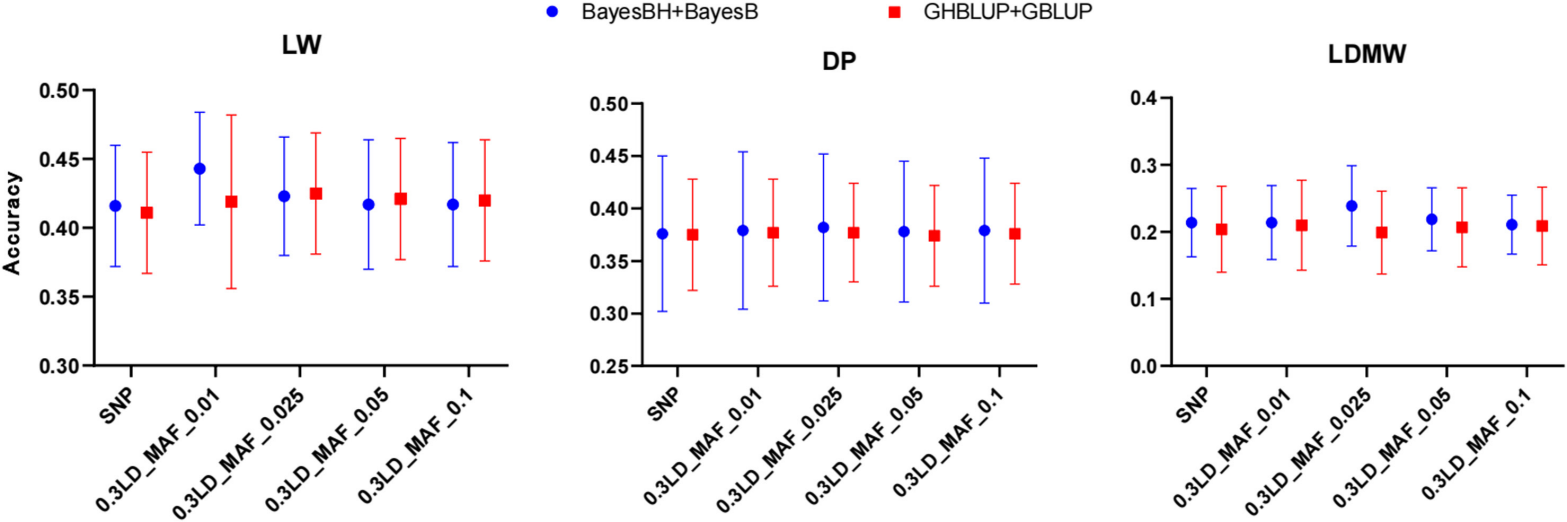
Prediction accuracies of the SNP model and 0.3 LD haplotype model at different MAF thresholds. DP, dressing percentage; LD, linkage disequilibrium; LDMW, longissimus dorsi muscle weight; LW, liveweight; MAF, minor allele frequency; SNP, single nucleotide polymorphisms.

**FIGURE 4:**
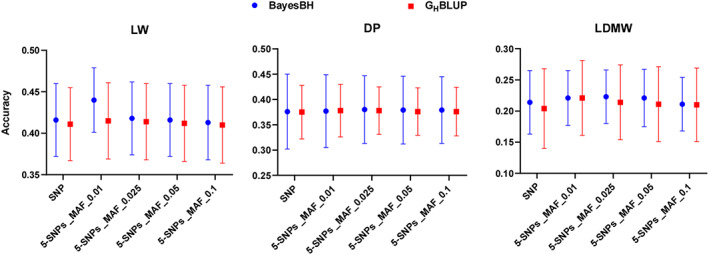
Prediction accuracies of the SNP model and 5‐SNPs haplotype model at different MAF thresholds. DP, dressing percentage; LD, linkage disequilibrium; LDMW, longissimus dorsi muscle weight; LW, liveweight; MAF, minor allele frequency; SNP, single nucleotide polymorphisms.

To evaluate the prediction accuracy with different MHAF thresholds, the haplotype alleles frequency less than 0.01, 0.025, 0.05, and 0.1 were removed, respectively. The prediction performance of GP using four models, such as BayesBH+BayesB, G_H_BLUP+GBLUP, BayesBH, and G_H_BLUP were compared at different MHAF thresholds. For LW, 0.3LD and 5‐SNPs haplotype using BayesBH+BayesB model showed the highest when the MHAF threshold was greater than 0.01 (Figures 3 and 4; Tables S4 and S5). As for DP and LDMW, when MHAF was greater than 0.025, 0.3LD haplotype and 5‐SNPs haplotype using the BayesBH+BayesB model showed the highest accuracy. The accuracies improved by 1.05%–1.57% for DP and 4.04%–10.46% for LDMW, respectively. In addition, 0.3LD and 5‐SNPs haplotype method can reduce the computing time by 30.59%–31.6% and 9.64%–16.49%, compared with the SNP method (Tables S4 and S5).

### 3.3 Number of covariates and computation time of different models

The average number of covariates fitted in models for three traits and the corresponding computation time were shown in Table 6. In the study, both the number of covariates and computation time decreased with the LD (from *r* ^2^ > 0.2 to *r* ^2^ > 0.8) and the increase of the number of fixed‐SNPs (from 5 to 20). The computation time of GP using the LD‐based BayesBH+BayesB model was similar to that of the fixed‐SNPs BayesBH model. The computation time of the G_H_BLUP+GBLUP model was slightly larger than G_H_BLUP, and GBLUP model due to long time was required to calculate the covariance of the two G matrixes in the G_H_BLUP+GBLUP model. Because the MCMC sampling often takes at least an order of magnitude, the Bayesian models cost much more time to fit covariates compared with the GBLUP models (Table 6). The Bayesian haplotype methods have fewer covariates than the SNP methods with 1% SNP allele frequency filter. The LD‐based BayesBH+BayesB model can reduce by 29.3% of the average computation time compared with the BayesB SNP model. However, the accuracy of BayesBH+BayesB models was larger than the BayesB model in most scenarios (Figure 2 and Table 5). It is worth mentioning that the LD‐based BayesBH model can drastically reduce the computation time because the few covariates were fitted in the model (Table 6), while the accuracy of GP for LW using the LD‐based BayesBH model is higher than that of the BayesB model (Figure 2 and Table 5).

**TABLE 6:** Computation time and number of covariates in different models

Method	*r* ^2^ ^a^ /Number of SNPs^b^	Computation time (h)^c^						Number of covariates^d^					
		GBLUP	G_H_BLUP	G_H_+G	BayesB	BayesBH	BH+B	GBLUP	G_H_BLUP	G_H_+G	BayesB	BayesBH	BH+B
SNP	—	0.006	—	—	19.41	—	—	672,060	—	—	672,060	—	—
LD	0.2	—	0.025	0.05	—	5.37	14.425	—	691,884	1,010,588	—	280,575	598,160
	0.3	—	0.022	0.05	—	4.23	13.71	—	543,155	914,760	—	235,534	605,908
	0.4	—	0.02	0.054	—	4.02	14.16	—	444,887	856,417	—	200,621	610,845
	0.5	—	0.024	0.057	—	3.27	13.23	—	373,007	817,085	—	172,198	614,902
	0.6	—	0.02	0.062	—	2.7	13.59	—	316,566	788,801	—	148,697	619,493
	0.7	—	0.025	0.058	—	2.4	13.56	—	268,438	766,424	—	127,200	623,674
	0.8	—	0.024	0.06	—	1.83	13.38	—	223,688	747,477	—	105,810	628,034
Fix‐SNPs	5	—	0.031	—	—	19.73	—	—	755,697	—	—	755,697	—
	10	—	0.033	—	—	17.93	—	—	642,861	—	—	642,861	—
	20	—	0.026	—	—	16.67	—	—	493,764	—	—	493,764	—

Abbreviations: Fixed‐SNPs, fixed‐SNPs Haploblock construction method; LD, LD‐based Haploblock construction method; LD, linkage disequilibrium; SNP, single nucleotide polymorphisms.

^a^ The seven different LD thresholds set from *r* ^2^ > 0.2 to *r* ^2^ > 0.8 to construct LD‐based haploblocks.

^b^ The three levels of number of SNPs (5, 10, and 20) to construct fixed‐SNP haploblocks.

^c^ Computation time for running the analysis based on GBLUP and Bayesian model with a chain length of 30,000.

^d^ The number of SNPs or haplotype alleles fitted in models.

## 4 DISCUSSION

The genomic selection has been widely applied to many farm animals (Meuwissen et al., 2001). Various methods based on the SNPs were used to improve the accuracies of GP (Gianola, 2013; VanRaden, 2008; Yin et al., 2020). Haploblock comprising a set of neighboring SNPs (likely to be inherited together) has a higher LD with quantitative trait loci (QTLs) than SNPs, thus GP method integrating haplotype should be effective to estimate the effect of QTLs and can further improve the prediction accuracies. Our study evaluated the performance of GP using several models (BayesBH+BayesB, G_H_BLUP+GBLUP, BayesBH, and G_H_BLUP) based on the different haplotype construction methods in beef cattle. We found the haplotype‐based GP generated better performance compared with SNPs, which was consistent with previous studies (Cuyabano et al., 2014; Cuyabano, Su, & Lund, 2015; Hess et al., 2017; Li et al., 2021). In addition, GP based on haplotype was an effective method to reduce the number of variables and compress the computation time, thus this method is beneficial for GP using whole‐genome sequence data.

### 4.1 Analysis of haploblock structure using different construction methods

We found the haploblock structure showed diverse patterns using different haplotype construction methods based on LD and fixed number of SNPs. The number of haploblocks decreased under thresholds with LD (from *r* ^2^ > 0.2 to *r* ^2^ > 0.8) and the number of fixed‐SNPs (from 5‐SNPs to 20‐SNPs). These results were similar to previous studies (Hess et al., 2017). We also observed that the number of haplotype alleles (covariates) using fixed‐SNP methods was larger than that of LD‐based methods (Table 6), this can be explained that the fixed‐SNP method used all the SNPs thus it produced more haplotype alleles. Moreover, the previous study presented that variable‐length haploblocks can reflect the true haploblock structure compared with fixed‐length haploblocks due to different LD levels among populations, which cannot be reflected by fixed‐length haploblocks (Hess et al., 2017).

### 4.2 Haplotype‐based GP using different construction methods

We evaluated the performance of GP for three traits (with heritability ranging from 0.16 to 0.40) based on diverse LD levels and different numbers of fixed‐SNPs. We found that the haplotype methods can produce high accuracies across the three traits in most scenarios when LD thresholds were *r* ^2^ > 0.3 or *r* ^2^ > 0.4 and the number of fixed‐SNPs was five (Figures 1 and 2; Tables 4 and 5). As shown in Table 6, the average number of SNPs per haploblock was 4.64 and 4.31 under the thresholds *r* ^2^ > 0.3 or *r* ^2^ > 0.4. Therefore, the haplotype structure using two construction methods may be similar under *r* ^2^ > 0.3 or *r* ^2^ > 0.4 and the number of fixed‐SNPs was five (Table 6). Cuyabano et al. (2014) reported that the LD‐based haploblocks can lead to the highest accuracies for milk protein, fertility, and mastitis traits when $D^{\prime }\geq 0.45$. Villumsen and Janss (2009) also revealed the 5‐SNP method can produce the highest prediction accuracy when GP based on haplotypes were investigated in simulated data. In addition, Hess et al. (2017) found the short haploblock (250 kb) can generate an optimal set of variables in admixed dairy cattle population, and the average number of SNPs per haploblock in 250 kb haplotype was four, which was similar to our study.

In this study, the LD‐based methods showed slightly higher accuracy under some thresholds compared with the accuracies of GP using fixed‐SNP methods. One possible explanation is that LD‐based haploblocks can reflect the true block structure of the population compared with fixed‐SNPs haploblocks. However, LD‐based methods did not consistently show improvement over SNPs or fixed‐SNPs haplotype as expected for all three traits (Figure 1), which may be caused by the different genetic architectures among traits.

### 4.3 Performance of different GP models

The accuracies for GP using most haplotype methods are improved compared with the SNP methods in the current study. One possible explanation was the high LD between the haplotype alleles and linked QTLs (Zondervan & Cardon, 2004). Sun et al. (2013) showed that the effects of low‐MAF QTLs can be effectively captured by haplotype alleles. Moreover, the haplotype contained a group of SNPs, which may capture short‐range interaction among SNPs and improve the accuracies of GP (Kühn et al., 2004; Littlejohn et al., 2014). We also found that the BayesBH+BayesB models were robust and suitable for haplotype analyses in most scenarios. Similar to our results, the previous study reported that the Bayesian model with haploblocks and nonblocked SNPs can produce the highest accuracy for mastitis, which was the most unstable trait for genetic prediction (Cuyabano et al., 2014). The BayesBH+BayesB models included the haplotype alleles in blocks and SNP alleles outside the blocks. Many previous studies have proved that the Bayesian model can exploit LD information between SNPs and their linked QTLs compared with the GBLUP model (Gao et al., 2013; Habier et al., 2010; Ostersen et al., 2011). The advantage can further be amplified with Bayesian haplotype models due to the high LD between QTLs and haplotypes (Zondervan & Cardon, 2004). As for all three traits, the fixed‐SNP G_H_BLUP model has a high improvement for GP compared with the GBLUP model (Figure 2; Table 5). The reason can be explained that the true IBD relationship was captured by the haplotype alleles (combinations of phased SNP alleles) than the individual SNP alleles (Ferdosi et al., 2016; Hickey et al., 2013). Therefore, $G_{H}$ matrix improved the ability to detect ancestral relationships and led to higher accuracy of GP than G matrix. However, haploblock length can greatly influence the ability to capture IBD relationships, if the haploblock length was not suitable, they cannot reflect the true relationships between relatives (Ferdosi et al., 2016). Thus, the improvement of accuracy using the 20‐SNPs G_H_BLUP model was not significant for LW and DP compared with GBLUP (Figure 2; Table 5).

In the present study, haplotype‐based BayesBH methods have larger biases than the SNP‐based methods in most scenarios. The LD‐based methods were used to preselect SNPs with a certain degree of LD. The SNP effects in each of the haploblock may be overestimated due to “Beavis effect” (Xu, 2003), which indicates that the reported effects of SNPs tend to be larger than the true effects of them, which may lead to potential bias for GEBV (Zollner & Pritchard, 2007).

### 4.4 Number of covariates and computation time

The computation time for the BayesB model was more than the GBLUP model (Table 6). This was because that more time was required to fit SNPs in each round of a Markov‐chain Monte Carlo (MCMC) algorithm and estimated the SNP effects (Meuwissen et al., 2001). Our results also showed that the haplotype methods can produce more haplotype alleles with low frequency (rare haplotype alleles), especially for the long haploblocks (Tables 2 and 6). These rare haplotype alleles would affect prediction accuracy because the rare haplotype alleles are not possible to explain more genetic variance due to their low frequency, therefore the effect of rare alleles may be shrunk to zero (Gianola, 2013). The haplotype alleles with MHAF less than 0.01 were discarded in the fixed‐SNPs Bayesian haplotype model. We found the number of covariates of haplotype methods was reduced to 17%–50% for all haplotype alleles (Tables 2 and 6). The prediction accuracies of haplotype methods were slight decrease as a large number of low‐frequency haplotype alleles removed. This may be the potential reason why we observed the drop in the accuracy of GP for long haploblocks (i.e., 20‐SNPs haploblocks) (Figure 2).

### 4.5 Comparison of fixed‐SNPs and LD‐based haplotype

Two methods (fixed‐SNPs and LD‐based) were used to construct the haploblock, the LD‐based methods produce a high accuracy of GP compared with the fixed‐SNP methods (Tables S1 and S2). A previous study has reported that using methods by defining haploblocks with LD information (variable‐length haplotypes) can generate higher prediction accuracies than fixed‐length haploblocks (Hess et al., 2017), which was agreed with our findings. The LD between haplotype alleles and QTL were vary because of the recombination and artificial selection (Goddard et al., 2010). The optimal haploblock lengths may differ across the genome, thus application of variable‐length haploblock is likely to be an effective method that can contain the LD or recombination information. By contrast, the fixed‐SNP methods can not reveal the variation of LD. Many methods based on recombination (Heinemann, 2019), IBD probabilities (Calus et al., 2008, 2009), and smoothing spline techniques (Beissinger et al., 2015) were proposed to define the variable‐length haploblocks and further promote the improvement of GP. These methods can greatly enrich the methods of haplotype construction. The length of these haploblocks construction methods can vary along the genome of different populations.

### 4.6 Performance of GP with different MHAF

In this study, the optimal MHAF threshold for LW was set at 0.01, and the optimal MHAF threshold for DP and LDMW was set at 0.025. Under the optimal MHAF threshold, we found the accuracies of GP increased using the haplotype‐based method compared with the SNP method, while the computation time was reduced. Hess et al. (2017) suggested that haplotype‐based GP with the most frequent haplotype alleles can reduce computation time for short haplotypes in dairy cattle, while only a slight decrease in prediction accuracy was observed. Moreover, many studies showed that haplotype‐based GP with higher frequency was more accurate than that with lower frequency (Boleckova et al., 2012; Kolbehdari et al., 2007). By contrast, previously suggested that computation time was reduced with the little expected decrease in prediction accuracy by removing rare haplotype alleles because the effect of haplotype alleles is shrunk toward zero in Bayesian linear regression models (Gianola, 2013).

## 5 CONCLUSION

Our study showed that haplotype‐based Bayesian and GBLUP methods can improve the prediction accuracy up to 8.54% and 9.93% compared with the individual SNP approach. The LD‐based haplotype approach has larger improvements than that of fixed‐SNPs. When LD thresholds were *r* ^2^ > 0.3 or *r* ^2^ > 0.4 and the number of fixed‐SNPs was five, the Bayesian haplotype methods (BayesBH+BayesB) showed high accuracies across the three traits in most scenarios. Meanwhile, the average computation time of the LD‐based BayesBH+BayesB method can be reduced by 29.3% compared with the BayesB SNP method. LD‐based G_H_BLUP+GBLUP and fixed‐SNPs‐based G_H_BLUP methods using the 5‐SNPs can increase the accuracy of GP compared with GBLUP for all three traits. The optimal MHAF threshold for LW was 0.01, and the optimal MHAF threshold for DP and LDMW was 0.025. Further studies were required to evaluate the relationships within and between training and validation sets and further improve the accuracy of GP.

## FUNDING INFORMATION

This work was supported by the National Natural Science Foundation of China (31802049, 31872975, and 31972554), the Chinese Academy of Agricultural Sciences of Technology Innovation Project (CAAS‐ZDRW202102, CAAS‐XTCX2016010, CAAS‐ZDXT2018006 and ASTIP‐IAS03, ASTIP‐IAS‐TS‐16), the Beijing Natural Science Foundation (6154032), the National Beef Cattle Industrial Technology System (CARS‐37), the China Agriculture Research System of MOF and MARA, and the Science and Technology Project of Inner Mongolia Autonomous Region (2020GG0210).

## CONFLICT OF INTEREST

The authors declare that they have no competing interests.

## Supporting information


Table S1

Table S2

Table S3

Table S4

Table S5

## Data Availability

Partial genotype data have been submitted to Dryad: doi:10.5061/dryad.4qc06. The phenotype data used during the current study are available from the corresponding author upon reasonable request after the manuscript is accepted for publication.
