# Supplementary material for: Genomic prediction of carcass traits using different haplotype block partitioning methods in beef cattle
Source: Evol Appl. 2022 Nov 14;15(12):2028–42. doi: 10.1111/eva.13491 (PMC9753827; doi:10.1111/eva.13491)
Supplement: Supplementary file 1 — Table S1 [file EVA-15-2028-s005.docx]

**Table S1** Predictive Accuracies and Bias using LD-based Haplotype Model for 3 traits in Chinese Simmental beef cattle (±SD)

LW=Liveweight; DP=Dressing Percentage; LDMW= Longissimus Dorsi Muscle Weight

| ${\mathbf{r}^{\mathbf{2}}}^{\mathbf{1}}$ | **Model** | **LW** | | **DP** | | **LDMW** | |
| --- | --- | --- | --- | --- | --- | --- | --- |
|  |  | **ACC±SD** | **Bias±SD** | **ACC±SD** | **Bias±SD** | **ACC±SD** | **Bias±SD** |
| SNP | GBLUP | 0.412±0.043 | 1.02±0.109 | 0.376±0.048 | 1.033±0.174 | 0.196±0.062 | 0.953±0.409 |
|  | BayesB | 0.416±0.044 | 1.316±0.185 | 0.376±0.074 | 1.678±0.394 | 0.214±0.051 | 1.192±0.377 |
| 0.2 | G_H_BLUP | 0.407±0.047 | 1.009±0.118 | 0.376±0.047 | 1.04±0.175 | 0.209±0.062 | 0.963±0.386 |
| 0.3 | G_H_BLUP | 0.407±0.046 | 1.011±0.116 | 0.374±0.048 | 1.038±0.179 | 0.202±0.062 | 0.966±0.397 |
| 0.4 | G_H_BLUP | 0.405±0.046 | 1.01±0.121 | 0.376±0.05 | 1.035±0.182 | 0.202±0.062 | 0.967±0.398 |
| 0.5 | G_H_BLUP | 0.404±0.046 | 1.011±0.121 | 0.373±0.05 | 1.029±0.184 | 0.204±0.062 | 0.968±0.39 |
| 0.6 | G_H_BLUP | 0.405±0.044 | 1.012±0.116 | 0.372±0.051 | 1.028±0.186 | 0.2±0.061 | 0.971±0.39 |
| 0.7 | G_H_BLUP | 0.401±0.044 | 1.012±0.119 | 0.371±0.051 | 1.029±0.189 | 0.203±0.058 | 0.98±0.377 |
| 0.8 | G_H_BLUP | 0.397±0.043 | 1.009±0.117 | 0.368±0.05 | 1.024±0.187 | 0.207±0.054 | 0.981±0.344 |
| 0.2 | BayesBH | 0.438±0.039 | 1.227±0.206 | 0.369±0.073 | 1.777±0.406 | 0.205±0.043 | 1.249±0.387 |
| 0.3 | BayesBH | 0.438±0.041 | 1.202±0.176 | 0.373±0.074 | 1.724±0.397 | 0.206±0.052 | 1.238±0.42 |
| 0.4 | BayesBH | 0.439±0.042 | 1.202±0.181 | 0.376±0.071 | 1.711±0.375 | 0.211±0.047 | 1.397±0.44 |
| 0.5 | BayesBH | 0.438±0.04 | 1.227±0.185 | 0.372±0.073 | 1.78±0.41 | 0.212±0.048 | 1.445±0.447 |
| 0.6 | BayesBH | 0.439±0.041 | 1.229±0.179 | 0.372±0.074 | 1.869±0.452 | 0.204±0.046 | 1.315±0.406 |
| 0.7 | BayesBH | 0.412±0.045 | 1.38±0.198 | 0.368±0.075 | 1.887±0.479 | 0.212±0.046 | 1.603±0.508 |
| 0.8 | BayesBH | 0.411±0.046 | 1.456±0.22 | 0.369±0.073 | 1.967±0.487 | 0.21±0.047 | 1.408±0.443 |
| 0.2 | G_H_BLUP +GBLUP | 0.403±0.068 | 0.982±0.152 | 0.377±0.044 | 1.038±0.164 | 0.2±0.067 | 0.948±0.422 |
| 0.3 | G_H_BLUP +GBLUP | 0.412±0.08 | 1.071±0.118 | 0.378±0.043 | 1.024±0.171 | 0.201±0.066 | 0.946±0.415 |
| 0.4 | G_H_BLUP +GBLUP | 0.417±0.056 | 1.009±0.128 | 0.376±0.045 | 1.035±0.166 | 0.2±0.065 | 0.945±0.41 |
| 0.5 | G_H_BLUP +GBLUP | 0.42±0.053 | 1.008±0.124 | 0.376±0.045 | 1.034±0.167 | 0.199±0.065 | 0.948±0.412 |
| 0.6 | G_H_BLUP +GBLUP | 0.422±0.052 | 1.001±0.126 | 0.376±0.045 | 1.021±0.176 | 0.199±0.065 | 0.949±0.412 |
| 0.7 | G_H_BLUP +GBLUP | 0.424±0.048 | 1.014±0.121 | 0.374±0.045 | 1.019±0.178 | 0.199±0.064 | 0.95±0.411 |
| 0.8 | G_H_BLUP +GBLUP | 0.42±0.048 | 1.011±0.12 | 0.374±0.047 | 1.034±0.174 | 0.198±0.064 | 0.951±0.412 |
| 0.2 | BayesBH+BayesB | 0.44±0.045 | 1.297±0.187 | 0.377±0.075 | 1.644±0.377 | 0.215±0.049 | 1.203±0.37 |
| 0.3 | BayesBH+BayesB | 0.443±0.041 | 1.23±0.189 | 0.379±0.075 | 1.641±0.381 | 0.214±0.055 | 1.146±0.392 |
| 0.4 | BayesBH+BayesB | 0.439±0.039 | 1.202±0.196 | 0.376±0.075 | 1.646±0.385 | 0.232±0.057 | 0.978±0.41 |
| 0.5 | BayesBH+BayesB | 0.421±0.044 | 1.307±0.181 | 0.374±0.074 | 1.668±0.393 | 0.231±0.055 | 1.019±0.454 |
| 0.6 | BayesBH+BayesB | 0.419±0.041 | 1.249±0.197 | 0.376±0.076 | 1.688±0.403 | 0.211±0.049 | 1.227±0.387 |
| 0.7 | BayesBH+BayesB | 0.424±0.012 | 1.342±0.185 | 0.374±0.074 | 1.691±0.4 | 0.212±0.05 | 1.175±0.371 |
| 0.8 | BayesBH+BayesB | 0.419±0.042 | 1.318±0.177 | 0.378±0.075 | 1.668±0.398 | 0.212±0.052 | 1.234±0.403 |

1) The seven different LD thresholds set from $r^{2}>0.2$ to $r^{2}>0.8$ to construct LD-based haploblocks.

.
