# Supplementary material for: Genomic prediction of carcass traits using different haplotype block partitioning methods in beef cattle
Source: Evol Appl. 2022 Nov 14;15(12):2028–42. doi: 10.1111/eva.13491 (PMC9753827; doi:10.1111/eva.13491)
Supplement: Supplementary file 2 — Table S2 [file EVA-15-2028-s004.docx]

**Table S2** Predictive Accuracies and Bias using FixedSNPs-based Haplotype Model for 3 traits in Chinese Simmental beef cattle (±SD)

LW=Liveweight; DP=Dressing Percentage; LDMW= Longissimus Dorsi Muscle Weight

| **Number** | **Model** | **LW** | | **DP** | | **LDMW** | |
| --- | --- | --- | --- | --- | --- | --- | --- |
| **FixedSNPs^1^** |  | **ACC±SD** | **Bias±SD** | **ACC±SD** | **Bias±SD** | **ACC±SD** | **Bias±SD** |
| SNP | GBLUP | 0.412±0.043 | 1.02±0.109 | 0.376±0.048 | 1.033±0.174 | 0.196±0.062 | 0.953±0.409 |
|  | BayesB | 0.416±0.044 | 1.316±0.185 | 0.376±0.074 | 1.678±0.394 | 0.214±0.051 | 1.192±0.377 |
| 5 | G_H_BLUP | 0.417±0.047 | 1.031±0.12 | 0.379±0.046 | 1.05±0.171 | 0.217±0.061 | 0.969±0.356 |
|  | BayesBH | 0.44±0.039 | 1.168±0.17 | 0.377±0.072 | 1.627±0.355 | 0.221±0.044 | 1.236±0.346 |
| 10 | G_H_BLUP | 0.417±0.048 | 1.039±0.128 | 0.377±0.045 | 1.03±0.168 | 0.221±0.062 | 1.051±0.401 |
|  | BayesBH | 0.42±0.044 | 1.288±0.19 | 0.374±0.071 | 1.574±0.34 | 0.222±0.047 | 1.401±0.408 |
| 20 | G_H_BLUP | 0.415±0.048 | 1.068±0.136 | 0.376±0.046 | 0.986±0.165 | 0.219±0.059 | 1.289±0.52 |
|  | BayesBH | 0.419±0.045 | 1.323±0.202 | 0.373±0.069 | 1.583±0.337 | 0.221±0.048 | 1.218±0.362 |

1) The three levels of number of SNPs (5, 10 and 20) to construct fixedSNP-based haploblocks.
