# Supplementary material for: Genomic prediction of carcass traits using different haplotype block partitioning methods in beef cattle
Source: Evol Appl. 2022 Nov 14;15(12):2028–42. doi: 10.1111/eva.13491 (PMC9753827; doi:10.1111/eva.13491)
Supplement: Supplementary file 3 — Table S3 [file EVA-15-2028-s002.docx]

**Table S3** P-values of the One-sided Paired t-test using Haplotype Model Rather than SNPs for 3 traits in Chinese Simmental beef cattle

| **Model^1^** | **Trait** | **LD-based method^2^** | | | | | | | **Fixed-SNPs method^3^** | | |
| --- | --- | --- | --- | --- | --- | --- | --- | --- | --- | --- | --- |
|  |  | **0.2** | **0.3** | **0.4** | **0.5** | **0.6** | **0.7** | **0.8** | **5** | **10** | **20** |
| G_H_BLUP | LW | 0.687 | 0.412 | 0.526 | 0.415 | 0.138 | 0.223 | 0.403 | 0.025 | 0.081 | 0.379 |
|  | DP | 0.35 | 0.601 | 0.91 | 0.929 | 0.994 | 0.537 | 0.795 | 0.009 | 0.379 | 0.845 |
|  | LDMW | 0.011 | 0.029 | 0.041 | 0.075 | 0.189 | 0.144 | 0.012 | 0.003 | 0.001 | 0.005 |
| BayesBH | LW | 0.000 | 0.000 | 0.000 | 0.000 | 0.000 | 0.058 | 0.028 | 0.000 | 0.206 | 0.477 |
|  | DP | 0.011 | 0.139 | 0.81 | 0.118 | 0.034 | 0.000 | 0.004 | 0.711 | 0.46 | 0.369 |
|  | LDMW | 0.309 | 0.176 | 0.52 | 0.551 | 0.002 | 0.696 | 0.212 | 0.122 | 0.198 | 0.203 |
| G_H_+G | LW | 0.371 | 0.665 | 0.504 | 0.288 | 0.076 | 0.014 | 0.051 | - | - | - |
|  | DP | 0.55 | 0.291 | 0.981 | 0.956 | 0.865 | 0.338 | 0.369 | - | - | - |
|  | LDMW | 0.039 | 0.002 | 0.006 | 0.009 | 0.003 | 0.006 | 0.006 | - | - | - |
| B_H_+B | LW | 0.000 | 0.000 | 0.000 | 0.865 | 0.13 | 0.149 | 0.000 | - | - | - |
|  | DP | 0.552 | 0.026 | 0.669 | 0.017 | 0.795 | 0.076 | 0.104 | - | - | - |
|  | LDMW | 0.909 | 0.921 | 0.015 | 0.008 | 0.105 | 0.393 | 0.137 | - | - | - |

1) G_H_+G: G_H_BLUP+GBLUP Model; B_H_+B: BayesBH+BayesB Model

2) The seven different LD thresholds set from $r^{2}$>0.2 to $r^{2}$>0.8 to construct LD-based haploblocks.

3) The three levels of number of SNPs (5, 10 and 20) to construct fixed-SNP haploblocks.
