# Supplementary material for: Genomic prediction of carcass traits using different haplotype block partitioning methods in beef cattle
Source: Evol Appl. 2022 Nov 14;15(12):2028–42. doi: 10.1111/eva.13491 (PMC9753827; doi:10.1111/eva.13491)
Supplement: Supplementary file 4 — Table S4 [file EVA-15-2028-s003.docx]

**Table S4** Prediction accuracies of SNP model and 0.3LD haplotype model at different MAF thresholds (±SD)

| **MHAF** | **Item** | **LW** | | **DP** | | **LDMW** | |
| --- | --- | --- | --- | --- | --- | --- | --- |
|  |  | **B_H_+B^1^** | **G_H_+G^2^** | **B_H_+B** | **G_H_+G** | **B_H_+B** | **G_H_+G** |
| **SNP** | **ACC** | 0.416±0.044 | 0.411±0.044 | 0.376±0.074 | 0.375±0.053 | 0.214±0.051 | 0.204±0.064 |
|  | **Bias** | 1.316±0.185 | 1.016±0.115 | 1.678±0.394 | 1.029±0.196 | 1.192±0.377 | 0.997±0.424 |
|  | **Computation time (h)** | 19.41±1.289 | 0.006±0.001 | 18.68±1.156 | 0.008±0.002 | 18.76±1.336 | 0.0012±0.001 |
| **0.3LD_MAF_0.01^3^** | **Accuracy** | 0.443±0.041 | 0.419±0.063 | 0.379±0.075 | 0.377±0.051 | 0.214±0.055 | 0.21±0.067 |
|  | **Bias** | 1.23±0.189 | 1.071±0.118 | 1.641±0.381 | 1.016±0.191 | 1.146±0.392 | 0.944±0.367 |
|  | **Computation time (h)** | 13.71±0.954 | 0.05±0.03 | 13.96±0.892 | 0.047±0.03 | 13.44±0.871 | 0.042±0.07 |
| **0.3LD_MAF_0.025** | **Accuracy** | 0.423±0.043 | 0.425±0.044 | 0.382±0.07 | 0.377±0.047 | 0.239±0.06 | 0.199±0.062 |
|  | **Bias** | 1.321±0.185 | 1.012±0.116 | 1.326±0.286 | 1.024±0.169 | 0.957±0.353 | 0.914±0.387 |
|  | **Computation time (h)** | 12.115±0.671 | 0.035±0.02 | 12.965±2.298 | 0.052±0.05 | 12.831±0.74 | 0.056±0.033 |
| **0.3LD_MAF_0.05** | **Accuracy** | 0.417±0.047 | 0.421±0.044 | 0.378±0.067 | 0.374±0.048 | 0.219±0.047 | 0.207±0.059 |
|  | **Bias** | 1.339±0.194 | 1.007±0.119 | 1.347±0.281 | 1.022±0.172 | 1.225±0.356 | 0.983±0.376 |
|  | **Computation time (h)** | 9.2±3.96 | 0.042±0.04 | 11.025±1.329 | 0.051±0.05 | 12.22±1.793 | 0.058±0.023 |
| **0.3LD_MAF_0.1** | **Accuracy** | 0.417±0.045 | 0.42±0.044 | 0.379±0.069 | 0.376±0.048 | 0.211±0.044 | 0.209±0.058 |
|  | **Bias** | 1.342±0.19 | 1.006±0.122 | 1.362±0.291 | 1.021±0.171 | 1.216±0.351 | 0.978±0.387 |
|  | **Computation time (h)** | 8.375±2.175 | 0.041±0.06 | 9.663±1.972 | 0.069±0.07 | 9.867±1.676 | 0.028±0.018 |

1) G_H_+G: GHBLUP+GBLUP Model

2) B_H_+B: BayesBH+BayesB Model

3) 0.3LD_MAF_0.01: Haplotype alleles with a minor allele frequency less than 0.01 based on 0.3LD haplotype construction method were discarded
