# Supplementary material for: Genomic prediction of carcass traits using different haplotype block partitioning methods in beef cattle
Source: Evol Appl. 2022 Nov 14;15(12):2028–42. doi: 10.1111/eva.13491 (PMC9753827; doi:10.1111/eva.13491)
Supplement: Supplementary file 5 — Table S5 [file EVA-15-2028-s001.docx]

**Table S5** Prediction accuracies of SNP model and 5HAP haplotype model at different MAF thresholds (±SD)

| **MHAF** | **Item** | **LW** | | **DP** | | **LDMW** | |
| --- | --- | --- | --- | --- | --- | --- | --- |
|  |  | BayesBH | G_H_BLUP | BayesBH | G_H_BLUP | BayesBH | G_H_BLUP |
| **SNP** | **ACC** | 0.416±0.044 | 0.411±0.044 | 0.376±0.074 | 0.375±0.053 | 0.214±0.051 | 0.204±0.064 |
|  | **Bias** | 1.316±0.185 | 1.016±0.115 | 1.678±0.394 | 1.029±0.196 | 1.192±0.377 | 0.997±0.424 |
|  | **Computation time (h)** | 19.73±1.45 | 0.006±0.002 | 19.68±1.61 | 0.009±0.003 | 19.76±1.38 | 0.008±0.003 |
| **5HAP_MAF_0.01^1^** | **ACC** | 0.44±0.039 | 0.415±0.046 | 0.377±0.072 | 0.378±0.052 | 0.221±0.044 | 0.221±0.06 |
|  | **Bias** | 1.168±0.17 | 1.027±0.128 | 1.627±0.355 | 1.044±0.195 | 1.236±0.346 | 0.988±0.357 |
|  | **Computation time (h)** | 19.73±1.88 | 0.031±0.01 | 20.52±1.71 | 0.021±0.01 | 20.43±1.29 | 0.035±0.02 |
| **5HAP_MAF_0.025** | **ACC** | 0.418±0.044 | 0.414±0.046 | 0.38±0.067 | 0.378±0.047 | 0.223±0.043 | 0.214±0.06 |
|  | **Bias** | 1.292±0.18 | 1.019±0.115 | 1.328±0.271 | 1.03±0.171 | 1.102±0.296 | 1.068±0.41 |
|  | **Computation time (h)** | 17.441±1.398 | 0.0412±0.05 | 16.434±3.275 | 0.0423±0.04 | 17.855±3.321 | 0.0417±0.03 |
| **5HAP_MAF_0.05** | **ACC** | 0.416±0.044 | 0.412±0.046 | 0.379±0.067 | 0.376±0.047 | 0.221±0.046 | 0.211±0.06 |
|  | **Bias** | 1.29±0.186 | 1.013±0.115 | 1.299±0.259 | 1.025±0.173 | 1.101±0.314 | 1.075±0.425 |
|  | **Computation time (h)** | 13.442±2.029 | 0.049±0.05 | 13.064±2.369 | 0.0411±0.04 | 13.456±2.754 | 0.0447±0.09 |
| **5HAP_MAF_0.1** | **ACC** | 0.413±0.045 | 0.41±0.046 | 0.379±0.066 | 0.376±0.048 | 0.211±0.043 | 0.21±0.059 |
|  | **Bias** | 1.326±0.191 | 1.009±0.115 | 1.337±0.265 | 1.023±0.174 | 1.125±0.314 | 1.081±0.43 |
|  | **Computation time (h)** | 10.141±1.078 | 0.0419±0.05 | 9.824±1.353 | 0.0472±0.04 | 9.971±1.187 | 0.0414±0.01 |

1) 5HAP_MAF_0.01: Haplotype alleles with a minor allele frequency less than 0.01 based on 5HAP haplotype construction method were discarded.
